# What should be included in the assessment of laypersons’ paediatric basic life support skills? Results from a Delphi consensus study

**DOI:** 10.1186/s13049-018-0474-5

**Published:** 2018-01-18

**Authors:** Asbjørn Børch Hasselager, Torsten Lauritsen, Tim Kristensen, Cathrine Bohnstedt, Claus Sønderskov, Doris Østergaard, Martin Grønnebæk Tolsgaard

**Affiliations:** 1 Copenhagen Academy for Medical Education and Simulation (CAMES), Herlev Ringvej 75, 2730 Herlev, Denmark; 2 The University of Copenhagen, Nørregade 10, 1017 Copenhagen, Denmark; 3 Department of Paediatric Anaesthesia, The Juliane Marie Centre, Rigshospitalet University Hospital of Copenhagen, Blegdamsvej 9, 2100 Copenhagen, Denmark; 4 Department of Children and Adolescence Medicine, Herlev Hospital, Herlev Ringvej 75, 2730 Herlev, Denmark; 5 Department of Paediatrics and Adolescent Medicine, The Juliane Marie Centre, Rigshospitalet University Hospital of Copenhagen, Blegdamsvej 9, 2100 Copenhagen, Denmark; 6 RedMitBarn – FirstAiders, Rosenørns Alle 1, 1970 Frederiksberg C, Denmark; 7 Department of Obstetrics, The Juliane Marie Centre, Rigshospitalet University Hospital of Copenhagen, Blegdamsvej 9, 2100 Copenhagen, Denmark

**Keywords:** Paediatric basic life support, Assessment, Layperson, Training, Education, International consensus

## Abstract

**Background:**

Assessment of laypersons’ Paediatric Basic Life Support (PBLS) skills is important to ensure acquisition of effective PBLS competencies. However limited evidence exists on which PBLS skills are essential for laypersons. The same challenges exist with respect to the assessment of foreign body airway obstruction management (FBAOM) skills. We aimed to establish international consensus on how to assess laypersons’ PBLS and FBAOM skills.

**Methods:**

A Delphi consensus survey was conducted. Out of a total of 84 invited experts, 28 agreed to participate. During the first Delphi round experts suggested items to assess laypersons’ PBLS and FBAOM skills.

In the second round, the suggested items received comments from and were rated by 26 experts (93%) on a 5-point scale (1 = not relevant to 5 = essential). Revised items were anonymously presented in a third round for comments and 23 (82%) experts completed a re-rating. Items with a score above 3 by more than 80% of the experts in the third round were included in an assessment instrument.

**Results:**

In the first round, 19 and 15 items were identified to assess PBLS and FBAOM skills, respectively. The ratings and comments from the last two rounds resulted in nine and eight essential assessment items for PBLS and FBAOM skills, respectively. The PBLS items included: “Responsiveness”,” Call for help”, “Open airway”,” Check breathing”, “Rescue breaths”, “Compressions”, “Ventilations“, “Time factor” and “Use of AED”. The FBAOM items included: “Identify different stages of foreign body airway obstruction”, “Identify consciousness”, “Call for help”, “Back blows“, “Chest thrusts/abdominal thrusts according to age”, “Identify loss of consciousness and change to CPR”, “Assessment of breathing” and “Ventilation”.

**Discussion:**

For assessment of laypersons some PBLS and FBAOM skills described in guidelines are more important than others. Four out of nine of PBLS skills focus on airway and breathing skills, supporting the major importance of these skills for laypersons’ resuscitation attempts.

**Conclusions:**

International consensus on how to assess laypersons’ paediatric basic life support and foreign body airway obstruction management skills was established. The assessment of these skills may help to determine when laypersons have acquired competencies.

**Trial registration:**

Not relevant.

**Electronic supplementary material:**

The online version of this article (10.1186/s13049-018-0474-5) contains supplementary material, which is available to authorized users.

## Background

Laypersons who participate in adult basic life support training courses are more likely to provide bystander cardio-pulmonary resuscitation (CPR) [1, 2]. The same is believed to be true for paediatric basic life support (PBLS). Bystander CPR for children with cardiac arrest especially improves both survival and neurological outcomes [3–5].

Elaborate guidelines exist on how laypersons should respond to life threatening incidents requiring PBLS [6, 7]. However there is limited evidence regarding which competencies laypersons should acquire to provide effective PBLS, as well as how to assess these competencies. Consequently PBLS assessments have been extrapolated from guidelines or modifications of adult assessment instruments [8, 9]; but the validity of such extensions are not inherent when used for different groups [10].

Current PBLS training courses are often time-based and lack an assessment component but this does not ensure that course participants have actually acquired the skills necessary to provide high-quality PBLS. Hence, assessment of laypersons’ PBLS competence is essential to evaluate if and when participants have acquired the skills needed to provide effective PBLS. In turn, this would make it possible to conduct competency-based rather than time-based training, and, secondly, to improve learning by providing feedback to the students [11].

For these reasons, the European Resuscitation Council (ERC) and the American Heart Association (AHA) has requested the development of guidelines as a foundation for uniform testing in PBLS training and simulation research [11, 12]. However, existing PBLS assessment instruments are heterogeneous, developed for different types of first aid responders and highly influenced by local practices and guidelines [8, 13–15]. Furthermore, the skills needed for effective PBLS may differ between laypersons and health professionals, as well as between in-hospital and out-of-hospital settings [16]. There is no international consensus on what should be included in the assessment of laypersons’ PBLS skills. The same challenges exist with respect to the assessment of foreign body airway obstruction management (FBAOM) skills where the existing literature is limited and no assessment instrument is available.

A Delphi consensus study was conducted with the aim of establishing international consensus on essential items to assess layperson’s PBLS and FBAOM skills. These two sets of skills were selected as they represent components of resuscitation in an out-of-hospital setting where immediate action by laypersons is essential [6, 7].

## Methods

The Delphi consensus study was conducted from 2nd of November 2015 through 7th of March 2016.

**Fig. 1:**
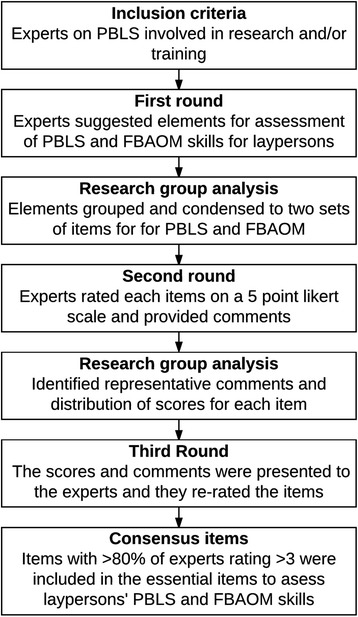
Consensus process flowchart. The figure illustrates the modified Delphi consensus process used in this study

We used a modified Delphi approach in order to seek consensus between experts in a number of consecutive rounds using structured questionnaires and a predefined consensus level [17, 18]. Experts remained anonymous throughout the process to avoid dominance by individual panel members [19]. The study was conducted in three consecutive rounds using email questionnaires (Fig. 1).

### Selection of experts

Experts were defined as health care professionals involved in training or research or both in paediatric resuscitation. They were identified from several sources: 1. Authors or contributors in the ERC 2015 paediatric guideline [7] or the Paediatric Consensus on Cardiopulmonary Resuscitation and Emergency Cardiovascular Care chapter [20] and experts from within the network of the research group.

### First round

An inductive approach with open-ended questions was selected for the first round. Experts were asked to suggest 3–15 important elements for the assessment of laypersons’ skills in PBLS and FBAOM. A reminder was sent to non-responders after one week. The elements suggested by the experts were grouped according to content and condensed into two sets of separate items with a short description for each item relating to either PBLS or FBAOM. The authors reviewed the grouping of the elements and the resulting condensed items. Disagreements were resolved by discussion until consensus was achieved.

### Second round

A questionnaire was sent to the experts who responded to the first round. The questionnaire included the condensed items from the first round with short descriptions for each. The presentation of findings was anonymous in all rounds. Experts were instructed to rate the items and encouraged to provide comments. Rating was done using a 5-point Likert scale with the anchors 1: Not relevant; 3: Relevant but not essential; and 5: Essential.

Participants received email reminders after one and three weeks.

The ratings and comments were analysed by the authors and descriptions were clarified based on comments from the experts. The distribution of ratings was established and mean values were calculated. The authors identified representative expert comments.

### Third round

Experts who responded in the second round were given a questionnaire, which included representative comments, item descriptions, mean values, and the distribution of ratings. They were asked to re-rate the items and to provide comments for the individual items based on the new information. All comments to the individual items from the second round were available in an appendix attached to the questionnaire. Participants received two reminders, after one and four weeks.

### Consensus definition

Consensus was sought for PBLS and FBAOM as two separate lists of items.

The criteria for inclusion in the final instruments for both sets of skills was defined a score above three by more than 80% of the experts in the third round [17, 21–23]. The experts were informed about these inclusion criteria prior to rating.

### Statistics

The Wilcoxon signed rank test was used to compare ratings (alpha level 0.05) from the last two Delphi rounds, in order to determine if experts changed their ratings.

All statistical analysis was performed using SAS studio 3.5 Copyright 2012–2016, SAS Institute Inc., Cary, NC, USA.

## Results

A total of 84 experts were invited of which 29 agreed to participate. One participant responded after deadline in the first round and was excluded. The participants included two nurses and 26 physicians of which 15 had an additional scientific degree (PhD, Professor or associate professor). They represented 13 countries with the following distribution of experts: Belgium (4), Brazil (1), Canada (1), Denmark (6), Germany (1), Hungary (1), Iceland (1), Portugal (1), Romania (1), Singapore (1), Spain (5), UK (2) and USA (3).

The suggested elements from the excluded participant would not have changed the condensed items.

Consensus was achieved after three rounds of rating. A total of 28, 26 and 23 participants completed each of the three rounds. The response rates in the second and third round were 92.9% and 82.1%, respectively.

Experts suggested 245 elements for PBLS skills and 189 for FBAOM in the first round with a median of 8 (Interquartile range 7–11) elements for PBLS and 6 (Interquartile range 5–8) for FBAOM. All suggested elements are listed in detail in an appendix (Additional file 1).

No new themes occurred after reading through the suggested elements proposed by the first 20 and 15 participants for PBLS and FBAOM skills, respectively.

The first round resulted in 19 items for PBLS (Table 1) and 15 items for FBAOM (Table 2) after the research group had reviewed, grouped and condensed the suggested elements**.**

**Table 1:** Grouped and condensed items for Paediatric Basic Life Support

Paediatric Basic Life Support										
Item number	Item	Description	Delphi round	Responses (n)	Distribution of likert ratings (%)					Wilcoxon signed-rank test (*p*-value)
					1	2	3	4	5	
1	Safety	Own safety and safety of the child	Second	26	4	12	8	15	62	0.24
			Third	23	0	13	22	22	44	
2	Responsiveness	Recognition of unresponsiveness	Second	26	0	0	12	27	62	0.56
			Third	23	0	0	0	35	65	
3	Call for help	Recognizing need for help and alerting *surroundings both by loud verbal call out and using telephone*	Second	26	0	0	8	23	69	>0.99
			Third	21	0	0	10	14	76	
4	Open airway	Ability to establish open airways including mouth inspection, appropriate head and jaw positioning.	Second	26	0	4	8	31	58	0.02
			Third	23	0	0	4	22	74	
5	Check breathing	Assessment of breathing and recognition of respiratory arrest *or abnormal breathing*	Second	26	0	0	15	46	39	0.73
			Third	23	0	0	9	65	26	
6	Rescue breaths	Ability to provide high quality *initial* rescue breaths	Second	25	0	4	0	40	56	0.56
			Third	23	0	0	4	35	61	
7	Compressions	High quality compressions. Adequate rate, compression depth and correct hand placement	Second	26	0	0	0	15	85	0.25
			Third	23	0	0	0	0	100	
8	Ventilations	High quality ventilations in general during CPR. Adequate thoracic rise.	Second	26	0	0	12	27	62	0.28
			Third	23	0	0	4	22	74	
9	Compression ventilation coordination	Deliver efficient compressions and ventilations in coordination	Second	26	0	8	15	62	15	0.31
			Third	21	0	5	24	57	14	
10	Call emergency medical service (EMS) / Communication with EMS	Ability to provide adequate information to emergency medical service by telephone	Second	26	4	8	15	23	50	0.27
			Third	21	0	10	38	29	24	
11	Two rescuers modifications	Ability to perform two rescuer CPR	Second	26	8	8	39	42	4	0.04
			Third	22	9	14	59	18	0	
12	Time factor	Ability to act effectively with minimised hands off time, no delays in treatment and fast call for help	Second	26	0	0	19	46	35	0.59
			Third	21	0	0	14	48	38	
13	Signs of life assessment	Ability to recognize signs of life to evaluate circulation	Second	25	0	8	20	52	20	0.82
			Third	23	0	9	26	52	13	
14	Recovery	Ability to recognize changes in condition *(*i.e. *return of spontaneous circulation) and act appropriately*	Second	26	8	8	27	54	4	0.40
			Third	22	5	9	59	27	0	
15	Adherence to algorithm	Ability to follow guidelines *and do procedures in the right sequence*	Second	26	0	8	39	39	15	0.03
			Third	22	0	14	68	18	0	
16	Non technical skills	Situational awareness, communication skills, use of available resources	Second	26	8	12	46	23	12	0.05
			Third	22	5	27	46	23	0	
17	Prevention of cardiac arrest	Recognize deteriorating conditions and call for help to prevent cardiac arrest	Second	25	0	16	20	28	36	0.68
			Third	23	0	13	30	39	17	
18	Allow for algorithm modification if not wanting to do ventilations	Appropriate action of continued compressions and call for help when not wanting or able to do ventilations	Second	26	8	12	31	31	19	0.06
			Third	23	4	17	48	30	0	
19	Use of AED	Appropriate use of and call for Automatic External defibrillator	Second	26	0	0	23	46	31	0.28
			Third	23	0	0	17	74	9	

The table shows the grouped and condensed items based on the participants responses in the first Delphi consensus round. Text in *italics* shows alterations in descriptions from the second to the third round

The table presents the distribution of ratings from the second and the third Delphi consensus round and Wilcoxon signed rank test of third round vs. second round distribution of scores for Paediatric Basic Life Support. Sum of percentages may not equal 100 due to rounding of percentages

**Table 2:** Grouped and condensed items for Foreign Body Airway Obstruction Management

Foreign Body Airway Obstruction Management										
Item number	Item	Description	Delphi round	Responses (n)	Distribution of likert ratings (%)					Wilcoxon signed-rank test (p-value)
					1	2	3	4	5	
1	Identify different stages of foreign body airway obstruction	Ability to distinguish effective and ineffective cough	Second	26	0	0	12	46	42	0.58
			Third	23	0	0	4	65	30	
2	Identify consciousness	Recognition of unresponsiveness	Second	26	4	0	4	27	65	0.12
			Third	23	0	0	0	13	87	
3	Call for help	Recognize need for help and alert surroundings	Second	26	0	0	4	27	69	0.75
			Third	23	0	0	9	17	74	
4	Back blows	High quality back blows with adequate force and correct place of impact	Second	26	4	0	0	35	62	0.49
			Third	22	0	5	9	18	68	
5	Chest thrusts/abdominal thrusts according to age	High quality chest thrust or abdominal thrust according to age	Second	26	0	0	4	31	65	0.16
			Third	22	0	0	0	27	73	
6	Identify loss of consciousness and change to CPR	Ability to recognize changes in condition and act appropriately	Second	26	0	0	4	15	81	0.66
			Third	23	0	0	0	17	83	
7	Non technical skills	Diagnose possible foreign body airway obstruction and act on prevention, situational awareness, communication skills, use of available resources	Second	26	4	12	31	35	19	0.13
			Third	23	4	13	52	30	0	
8	Adherence to algorithm / time factor	Ability to follow guidelines and minimise time delay in treatment	Second	26	0	4	27	58	12	0.3
			Third	22	0	9	32	59	0	
9	Assessment of effect and recovery position	Ability to recognise changes in condition (check mouth for foreign object between interventions) and act appropriately	Second	26	0	12	27	46	15	0.75
			Third	23	0	0	48	48	4	
10	Mouth inspection	Inspection and removal of visualized objects if confident item can be removed. *This item concerns active actions to remove foreign objects if confident they can be removed*	Second	25	0	12	28	32	28	0.43
			Third	23	0	13	35	39	13	
11	Airway	Adequate airway management (Altered in third round to) *Ability to position head according to age to open airway*	Second	24	8	0	17	29	46	0.52
			Third	22	14	9	14	14	50	
12	Assessment of breathing	Ability to assess breathing and recognition of respiratory arrest or abnormal breathing requiring ventilator support	Second	26	0	0	8	42	50	0.75
			Third	21	0	0	5	57	38	
13	Ventilation	Providing high quality ventilations *if patient stops breathing. Ability to provide ventilations with chest rise*	Second	25	4	4	0	36	56	0.26
			Third	22	0	0	5	32	64	
14	Modification of algorithm	Appropriate action of continued compressions and call for help when not wanting or able to do ventilations (if CPR is needed)	Second	26	12	12	27	35	15	0.38
			Third	23	9	9	48	26	9	
15	Complete/ incomplete obstruction	Ability to recognize complete or incomplete obstruction	Second	26	0	19	35	27	19	0.5
			Third	22	0	9	64	18	9	

The table shows the grouped and condensed items based on the participants’ responses in the first Delphi consensus round. Text in *italics* shows modifications in the descriptions from the second to the third round

The table presents the distribution of ratings from the second and the third Delphi consensus round and Wilcoxon signed rank test of third round vs. second round distribution of scores for Foreign Body Airway Obstruction Management. Sum of percentages may not equal 100 due to the rounding of percentages

The distribution of ratings for the second and third round for PBLS and for FBAOM is seen in Tables 1 and 2, respectively. Data was missing for 1% (7/884) of the item ratings in the second round and 3% (20/782) in third round.

In the first two rounds, the PBLS set of skills was referred to as “Paediatric cardiac arrest management for laypersons”. Expert comments from the second round suggested that the term “paediatric cardiac arrest management” could be misleading and experts might only consider verified cardiac arrest situations. To further emphasise that the intended target group was laypersons, the wording was changed from “paediatric cardiac arrest management” to the broader term “paediatric basic life support skills” which is consistent with the wording used by the ERC [7]. This modification was specifically mentioned in the third round questionnaire.

In the second round several expert provided comments indicating a lack of clarity in the descriptions of the following PBLS items: “3 - Call for help”, “5 - Check breathing”, “6 - Rescue breaths”, “14 - Recovery” and “15 - Adherence to algorithm” and of FBAOM items “10 - Mouth inspection”, “11 - Airway” and “13 - Ventilation”. The descriptions of these items were subsequently clarified during the third Delphi round. The text modifications were highlighted for the expert panel in the third round questionnaire and are marked in *italics* in Tables 1 and 2 for PBLS and FBAOM, respectively.

The item “15 - Adherence to algorithm” was the only item with a modified description that was scored significantly differently between the second and third rounds (*p* = 0.03). Three additional PBLS items scores changed significantly from the second to third round: “4 - Open airway” (*p* = 0.02), “11 - Two rescuers modifications” (*p* = 0.04) and “16 - Non technical skills” (*p* = 0.05).

There were no significant changes in scores for FBAOM items from round two to three.

Modification of item descriptions and changes in item scores did not affect the decision to include or exclude the items based on the predefined consensus criteria.

Eight PBLS items and eight FBAOM items scored above the predefined consensus level in the second round and were confirmed in the third round for inclusion in the final instrument to assess laypersons PBLS and FBAOM skills. One item “19 - Use of AED” was added to the final PBLS instrument after the third round. The resulting final assessment instrument items can be seen in Table 3 for PBLS and Table 4 for FBAOM.

**Table 3:** Final assessment instrument items for Paediatric Basic Life Support

Paediatric Basic Life Support	
Item	Description
Responsiveness	Recognise unresponsiveness
Call for help	Recognize need for help and alert surroundings both by loud verbal call out and using telephone
Open airway	Establish open airways including mouth inspection, appropriate head and jaw positioning
Check breathing	Assess breathing and recognize respiratory arrest or abnormal breathing
Rescue breaths	Provide high quality initial rescue breaths
Compressions	Provide high quality compressions. Adequate rate, compression depth and correct hand placement
Ventilations	Provide high quality ventilations in general during CPR with adequate chest rise
Time factor	Act effectively with minimised hands off time, no delays in treatment and fast call for help
Use of AED	Call for Automatic External defibrillator and appropriate use

The table summarizes the Paediatric Basic Life Support consensus items that fulfilled the consensus criteria for inclusion in the final instrument to assess laypersons’ skills after the third round. Description wordings have been aligned to ease the use. Content has not been changed

**Table 4:** Final assessment instrument items for Foreign Body Airway Obstruction Management

Foreign Body Airway Obstruction management	
Item	Description
Identify different stages of foreign body airway obstruction	Distinguish effective and ineffective cough
Identify consciousness	Recognize unresponsiveness
Call for help	Recognize need for help and alert surroundings
Back blows	Provide high quality back blows with adequate force and correct place of impact
Chest thrusts /abdominal thrusts according to age	Provide high quality chest thrust or abdominal thrust according to age
Identify loss of consciousness and change to CPR	Recognize changes in condition and act appropriately
Assessment of breathing	Assess breathing and recognize respiratory arrest or abnormal breathing requiring ventilator support
Ventilation	Provide high quality ventilations if patient stops breathing with adequate chest rise

The table summarises the Foreign Body Airway Obstruction Management consensus items that met the consensus level for inclusion in the final instrument to assess laypersons skills after the third round. Description wordings have been aligned to ease the use. Content has not been changed

## Discussion

This study established international consensus regarding the assessment of laypersons’ PBLS and FBAOM skills. Validity evidence was established and serves as a guide to what should be included in the assessment of laypersons’ PBLS and FBAOM skills.

Half of the PBLS consensus items (Table 3) are related to airway and breathing, which is more than in previous checklists, where airway and breathing related items constituted 29–35% of the items [8, 13, 14]. This indicates that the international consensus panel placed greater emphasis on airway and breathing skills, possibly due to the larger proportion of asphyxial cardiac arrests in paediatric patients [3, 7]. This focus is underscored by the inclusion of the items “12 - Assessment of breathing” and “13 - Ventilations” in FBAOM assessment (Table 4). Overall, these findings highlight the importance of viewing the two sets of skills (PBLS and FBAOM) as part of a continuum to improve survival.

The use of AED was identified as an essential skill in this study and is not found in other paediatric life support skills assessments. Both ERC and the AHA recommend use of AED in the event of sudden collapse [6, 7]. The emphasis on AED in this study may reflect the fact that AEDs are now more widespread and readily available. Other contributing factors could be that use of an AED is the only advanced life support treatment laypersons can offer, and the instructions given by these devices can help guide laypersons in resuscitation attempts with both shockable and non-shockable rhythms. On the other hand, it also important to consider that previous research suggests that AEDs can be used successfully with no training [24].

Although paediatric physiology does not differ across different geographic regions, health care systems and their settings do. Consequently, as all experts participating in this study represent developed world health care systems, the findings may not be able to be generalized to less developed countries. Nevertheless, this study demonstrates that it is feasible to establish consensus regarding generic content of an assessment instrument for PBLS and FBAOM skills. Future work should be directed towards local implementation. This could be accomplished by adding setting-specific components to the generic assessment items, although the validity of such additions has to be established.

Our findings suggest that there is a discrepancy between what guidelines describe as the ideal approach to PBLS in specified algorithms [6, 7] and what experts find essential for the assessment of laypersons’ PBLS skills. The final assessment items did not include directly identifiable guideline steps such as “1 – safety” and “15 - Adherence to algorithm”.

Secondly, we observed that the experts de-emphasised items dealing with very specific tasks such as “11 - Two rescuer modifications” and “18 - Allow for algorithm modification if not wanting to do ventilations”. The assessment items that were developed in this study emphasise general PBLS principles rather than detailed and specific tasks. This likely reflects the fact that laypersons are unlikely to ever encounter life-threatening events with children. The more general focus of the items also suggests that the instrument will be relevant across various guidelines and updates. However, in the future, the integration of new technologies in CPR efforts, such as telephone- and video dispatcher- assisted CPR, may result in the need for new skills related to the collaboration between provider and dispatcher [25].

Introducing assessment during PBLS courses for laypersons implies that some course participants will fail and need extended amount of training in order to achieve a satisfactory skill level. This may result in undesirable consequences if those who fail become discouraged from taking action in the event of a paediatric cardiac arrest. Studies of the effects of bystander CPR do not explore the quality of the bystander resuscitation attempt, but only its initiation and, consequently, they promote courses which improve the likelihood of bystander action [3–5]. However, if laypersons do not have sufficient skills, resuscitation attempts may not be successful and, thus, the courses will only improve participants’ confidence but not their ability to successfully resuscitate children with cardiac arrest or foreign body airway obstruction.

### Strength, limitations and future research

A potential bias of the Delphi methodology is that the research group may have had a significant impact on the generation and condensing of the proposed items. To compensate for this bias, we decided to include an open-ended questionnaire in the first round so that the expert panel, rather than the research group, generated the proposed items and saturation was achieved with the current sample of expert participants.

The significant changes in the distribution of scores, as well as the modifications in the descriptions and changes in the wording across the Delphi rounds, highlight the value of Delphi process as a method for establishing consensus. These findings suggest that the opinions of the expert panel were not immutable, but rather subject to change when confronted with the other experts’ comments and ratings [18].

The variation in the distribution of scores decreased for most items from the second to third round despite the fact that the expert group represented different countries and views on paediatric resuscitation. The experts not only agreed on the inclusion of essential content, but also on the exclusion of non-essential PBLS items. The observed shift in the scores during the final two rounds appears to represent progressive agreement regarding the perceived importance of various performance elements.

Although our participants were content experts with respect to paediatric resuscitation, they were not selected based on their experience with non-technical aspects of PBLS. Only a third of the invited expert participated, but they represented 13 different countries as well as different regions of the world. However, a majority of the included experts were from Western countries and few were from low-resource regions, which is a limitation for the generalization of results to these settings. In terms of size of the study, there is no agreement on the optimal number of experts to include in Delphi studies but achieving saturation is important. Although more than 12 participants are usually preferred [26].

Finally, we have established consensus on what to include in the assessment of laypersons’ PBLS and FBOAM skills, but we did not examine how well the resulting assessment instruments discriminate between providers with different levels of competence. Further studies are needed to determine the reliability and validity evidence of the PBLS and FBOAM assessment instruments, their use by raters and examinees, as well as their ability to discriminate between competent and non-competent laypersons [10].

## Conclusions

The study established international consensus on how to evaluate laypersons’ PBLS and FBAOM skills. The resulting assessment instruments may be used to determine when laypersons have attained the skills needed to provide effective paediatric resuscitation.

## Additional file

Additional file 1: Appendix with all suggested items by the participants. (DOCX 55 kb)

## Abbreviations

AHA: American Heart Association
CPR: Cardio-pulmonary resuscitation
ERC: European Resuscitation Council
FBAOM: Foreign body airway obstruction management
PBLS: Paediatric basic life support
