# Supplementary material for: What should be included in the assessment of laypersons’ paediatric basic life support skills? Results from a Delphi consensus study
Source: Scand J Trauma Resusc Emerg Med. 2018 Jan 18;26:9. doi: 10.1186/s13049-018-0474-5 (PMC5774155; doi:10.1186/s13049-018-0474-5)
Supplement: Additional file 1: — Appendix with all suggested items by the participants. (DOCX 55 kb) [file 13049_2018_474_MOESM1_ESM.docx]

# Appendix 1

## Legend

Suggested elements for assessment of Paediatric Basic Life Support and Foreign Body Airway Obstruction Management from the first Delphi round.

| **no** | **Suggested elements: Paediatric Basic Life Support** |
| --- | --- |
| 1 | Need to recognise cardiac arrest includes agonal breathing and NOT just absence of breathing |
| 2 | Emphasise NO need for pulse check |
| 3 | Emphasise good "chest compression" - "chest depth", "correct rate", "correct landmark" & "good recoil" - and use of evidence-based feedback devices in the teaching |
| 4 | Teach/allow chest-compression only CPR if unable to do ventilation (for whatever reasons) |
| 5 | If single rescuer and not familiar with CPR or CCR, to call for help first |
| 6 | Incorporate AED into teaching |
| 7 | Layperson updates/re-certification/reskilling process -- 2 to 3 yearly |
| 8 | Have a process to re-assess layperson's skills every 2 to 3 yearly |
| 9 | Use internet or app-based process for skills maintenance soon after layperson course |
| 10 | Teach good rescue breath techniques (again with feedback devices) to ensure adequate breaths delivered (not too much or too little) and good seal |
| 11 | Check for responsiveness |
| 12 | Call for help without leaving the child |
| 13 | Initiate resuscitation immediately |
| 14 | Open airway without excessive hyperextension |
| 15 | Ventilation to achieve adequate thoracic expansion |
| 16 | Evaluation of vital signs |
| 17 | Chest compressions rate 100 cpm |
| 18 | Chest compression with encircling technique in infants |
| 19 | Chest compression with one or two hands in children |
| 20 | Chest recoil time |
| 21 | Compression chest-ventilation coordination |
| 22 | Coordination chest compression-ventilation with two rescuers |
| 23 | BLS |
| 24 | correct ventilation (thorax movements) |
| 25 | Dephts of compressions |
| 26 | speed of compressions |
| 27 | landmarks |
| 28 | hands off time when AED is on |
| 29 | placements of pads |
| 30 | Hjertemassage |
| 31 | indblæsninger |
| 32 | Identificere hjertestop |
| 33 | Kalde hjælp |
| 34 | Hjertestopalgoritme 15:2 (30:2) |
| 35 | Brug af hjertestarter |
| 36 | Overall adherence to algorithm |
| 37 | Correct evaluation of consciousness |
| 38 | Establishes free airway |
| 39 | Performs look-listen-feel |
| 40 | Number of rescue breaths w/ chest rise |
| 41 | ventilation:compression ratio and speed |
| 42 | chest rise w/ventilation |
| 43 | depth & release of compressions |
| 44 | time to call for help |
| 45 | time to 1st rescue breath |
| 46 | time to 1st compression |
| 47 | time to first AED rythm analysis |
| 48 | percentage of time with CPR interruptions starting from first compression |
| 49 | recognition of cardiac arrest |
| 50 | calling for context relevant help |
| 51 | correct cardiac compressions |
| 52 | correct ventilation |
| 53 | foreign body maneuvers |
| 54 | Awareness of situation |
| 55 | Initiation of some action as soon as possible |
| 56 | Safety actions |
| 57 | Airway opening skills |
| 58 | Composite chest compressions quality score |
| 59 | Individual components of chest compressions quality: a) rate |
| 60 | idem b) depth |
| 61 | Idem c) hand position |
| 62 | idem d) decompression |
| 63 | Quality of initial and ulterior ventilations |
| 64 | Quality of chest compressions/ventilations coordination and continuity |
| 65 | Quality of calling to EMS |
| 66 | Composite use of AED score |
| 67 | Individual components of AED use |
| 68 | Communication with EMS on scene |
| 69 | Push Hard (5 to 6cm depth for children and 4 to 5cm depth for infants) |
| 70 | Push Fast (100-120/min for infants and children) |
| 71 | Minimize interruption (no pause more than 4 seconds for change of rescuer, no pause more than 10 seconds for AED/rhythm assessment and shock, if suggested) |
| 72 | How to call DISPATCHER (911?) and to communicate the emergency |
| 73 | How to open the airway and deliver rescue breaths for children and infants |
| 74 | How to judge EFFECTIVE VENTILATION (movement of chest, air movement), more than an occasional gasp …..e.g. how to differentiate gasping and ineffective ventilation from effective ventilation. |
| 75 | Hand or Finger/thumb location on the chest |
| 76 | assessment of breathing |
| 77 | how to maintain a patent airway |
| 78 | how to deliver effective chest compressions |
| 79 | two and one person CPR |
| 80 | Ability to measure compression rate |
| 81 | Measure compression depth |
| 82 | Measure full chest recoil |
| 83 | Minimized interruptions |
| 84 | Quick, efficient rescue breaths |
| 85 | Quick, efficient determination of need for CPR |
| 86 | Assessment of agonal breathing (not sure how important that is in children, not much is know about that). I understand this is about “assessing skills of lay people to detect agonal breathing”. If you wish to assess this, you may have to develop a testing tool for agonal breathing. Can be done either on video or with actors (child actors?). |
| 87 | Opening airway (more difficult in small children compared to adults). Assessing opening of the airway: should it be “visually assessed” by the instructor or confirmed by a sensor in a manikin? |
| 88 | Assessment of consciousness (should it be the same as in adults? If possible, yes, because otherwise confusion with learning two techniques: adult and child) |
| 89 | You write “lay persons” but I am still questioning if truly lay people should learn two techniques: child and adult CPR. If you are targeting first responders that deal specifically with children, that may be different. But we may not call them “lay”. Therefore, there is an issue with defining the target group, I think. |
| 90 | If you target lay people, feeling for a pulse should probably not be taught and assessed. |
| 91 | Calling the EMS: should certainly be assessed. Should the lay person find a phone or is it enough to say “I call the EMS”? Should the lay person “make” the call? Should the lay person be tested in a situation where he/she needs to leave the patient to make the call? |
| 92 | Should the lay person be tested in a telephone CPR situation? |
| 93 | Responsivness |
| 94 | Call for help |
| 95 | Breathing ? |
| 96 | rescue breaths |
| 97 | signs of life |
| 98 | compressions |
| 99 | ventilation |
| 100 | call emergency service |
| 101 | use of AED |
| 102 | kommunicatipon with 112 |
| 103 | transition between ventilation and compression |
| 104 | Check of responsiveness (e.g. AVPU) |
| 105 | Call for help |
| 106 | Check & opening of airway |
| 107 | Check of breathing |
| 108 | Rescue breaths (effectiveness) |
| 109 | Check for signs of life |
| 110 | Chest compressions (quality: site, depth, recoil, rate, no-flow-time) |
| 111 | Emergency call |
| 112 | Coordinated hand- and take-over to EMT |
| 113 | Check for AED availability |
| 114 | Communication |
| 115 | Team building |
| 116 | Use of resources |
| 117 | ADHERENCE TO THE SEQUENCE |
| 118 | CALLING FOR HELP |
| 119 | DELIVERY OF BREATHS IN THE RIGHT PLACE |
| 120 | GOING FOR HELP IF IT IS NOT ON ITS WAY |
| 121 | CORRECT COMPRESSION RATE |
| 122 | CORRECT COMPRESSION DEPTH |
| 123 | TIME FOR CHEST WALL RELAXATION |
| 124 | THE LAYPERSON CAN TEACH SOMEONE ELSE!- FACE TO FACE or ON THE PHONE |
| 125 | Prevention - recognition of the sick/pre-arrest child (difficulty in or abnormal breathing, change in responsiveness and/or conscious level |
| 126 | Recognition of the unresponsive child |
| 127 | Call for help |
| 128 | Opening the airway |
| 129 | Maintenance of an open airway |
| 130 | Provision of rescue breaths (where appropriate) or moving directly to chest compressions |
| 131 | Maintenance of the airway and breathing |
| 132 | Provision of chest compressions |
| 133 | Providing chest compressions and ventilations in an appropriate sequence until help arrives |
| 134 | Use of an AED if accessible and appropriate |
| 135 | If the rescuer does not want to perform mouth-to-mouth ventilation then they should proceed immediately to chest compressions and perform these continuously until help arrives |
| 136 | Assessment airway - head tilt, chin lift |
| 137 | Assessment breathing - movement of the chest wall during inspiration |
| 138 | Assessment chest compressions - adequate depth and rate |
| 139 | Ability to recognize unresponsiveness. |
| 140 | Ability to shout for help. |
| 141 | Ability to perform a jaw thrust. |
| 142 | Ability to recognise absence of respiration or presence of gasping. |
| 143 | Ability to perform mouth-to-mouth or mouth-to-nose and mouth breaths. |
| 144 | Ability to perform chest compressions at 100-120/minute. |
| 145 | Ability to avoid interruptions of chest compressions over 10 seconds. |
| 146 | Ability to call emergency response system. |
| 147 | establish safety |
| 148 | open airway |
| 149 | provide adequate rescue breaths |
| 150 | chest compression depth |
| 151 | chest compression rate |
| 152 | chest compression recoil |
| 153 | hand position |
| 154 | hands-off time |
| 155 | establish cardiac arrest |
| 156 | implement AED |
| 157 | 15:2 |
| 158 | implement 2-rescuer strategy |
| 159 | call 112 |
| 160 | call for help |
| 161 | Recognition of unresponsive child |
| 162 | Compression efficacy- depth |
| 163 | Compression efficacy - rate |
| 164 | Mouth to mouth ventilation |
| 165 | Recognition of signs of life (ROSC) |
| 166 | AED use |
| 167 | Activation of emergency services |
| 168 | Assessment of absence of signs of life |
| 169 | Opening airway according to the age |
| 170 | Giving breath mouth-to mouth/mouth+nose |
| 171 | Giving chest compressions according to the age |
| 172 | Recognizing of recovery of patient's signs of life |
| 173 | Recognition of loss of consciousness |
| 174 | Recognition of respiratory arrest |
| 175 | Recognition of cardiac arrest |
| 176 | When to ask for help |
| 177 | How to ask for help |
| 178 | How to open the airway |
| 179 | Breath support : Mouth to mouth and nose technique / mouth to mouth technique |
| 180 | How to recognize signs of life |
| 181 | How to perform chest compressions in infants |
| 182 | How to perform chest compressions in children |
| 183 | How to place the child in the recovery position |
| 184 | Ability to determine absence of response ​ |
| 185 | ​ Ability to determine "not breathing"​ |
| 186 | ​ Ability to identify gasping​ |
| 187 | ​ Ability to identify rescuer safety issues |
| 188 | ​ Good quality chest compressions​ (location, rate, depth) |
| 189 | ​ Ability to adequately transmit information to dispatch /EMS  ​ |
| 190 | ​ Ability to maintain correct safety position and adequately monitor child in that context |
| 191 | ​ Ability to correctly maintain patent airway and breathing (optional for lay persons?...)​ |
| 192 | Call for help |
| 193 | Adequate depth of compressions |
| 194 | Adequate rate of compressions |
| 195 | Calling for AED |
| 196 | Using an AED |
| 197 | Opening the airway |
| 198 | Rescue breathing |
| 199 | Safety |
| 200 | Recognition |
| 201 | Call EMS |
| 202 | 5 Rescue breaths |
| 203 | Chest compressions |
| 204 | 2 Ventilations |
| 205 | Use of AED |
| 206 | call for help |
| 207 | assessment (consciousness) |
| 208 | assessment (breathing) |
| 209 | assessment (signs of life) |
| 210 | open airway |
| 211 | rescue breathing |
| 212 | chest compression rate |
| 213 | chest compression depth |
| 214 | use of AED |
| 215 | safety (own and the victim) |
| 216 | RISK Situations awareness for cardiac arrest in children |
| 217 | open airway |
| 218 | establish respiratory arrest |
| 219 | breathing manoevre, mouth- mouth/nose and mask- mouth/nose |
| 220 | importance of rescue breaths |
| 221 | establish absence of signs of life |
| 222 | thoracic compressions/ratio |
| 223 | seek for help after one minute |
| 224 | incorporation of AED |
| 225 | Recognition of cardiac arrest |
| 226 | Context dependent call for help |
| 227 | No delay in start-up of CPR |
| 228 | CPR with no hands-off time |
| 229 | Evaluating ventilation effectiveness |
| 230 | Non-technical skills (team work, task management, situation awareness, decision making) |
| 231 | Check responsiveness |
| 232 | Open airway |
| 233 | check for breathing |
| 234 | Give rescue breaths |
| 235 | Check for sign of life |
| 236 | Give CPR |
| 237 | Call for help |
| 238 | Fast recognize a critically ill child |
| 239 | Asses vital parameters |
| 240 | Recognize correctly cardiac arrest |
| 241 | Begin CPR maneuver |
| 242 | Calling for help, knowing of emergency number |
| 243 | Placement of fingers, or hand/hands in chest compression |
| 244 | Depth and frequency of chest compression |
| 245 | Combine correctly chest compression with ventilation |

| **no** | **Suggested elements: Foreign Body Airway Obstruction management** |
| --- | --- |
| 1 | Create apps for "conscious" & "unconscious" choking child / choking infant and choking child - and the algorithms to manage accordingly |
| 2 | Conscious choking infant : Develop feedback devices and techniques for ensuring adequate chest compression and back thrusts in infants (both to ensure sufficient effort and to limit excessive efforts) |
| 3 | Conscious Choking Child : Develop feedback devices and technique to do adequate abdominal thrusts for unconscious choking child - landmarks, positioning of hands and direction of pressure etc |
| 4 | Unconscious choking infant/child - same as for above cardiac arrest, but emphasize check for visible FB to remove in the airway with adequate CPR - chest depth, chest rate, landmark & good recoil. |
| 5 | Unconscious choking child - emphasize re-checking for FB in airway after every cycle. |
| 6 | Diagnosis foreign body airway obstruction |
| 7 | Call for help |
| 8 | Finger extraction of foreign body |
| 9 | Adequate black blows in infants and children |
| 10 | Chest thrust in infants |
| 11 | Abdominal thrust in children |
| 12 | Check thoracic expansion with ventilation |
| 13 | Diagnosis of unresponsiveness |
| 14 | acknowledgement of consious and unconsious |
| 15 | landmarks for back blows, abdominal thrust and chest thrusts |
| 16 | open airways |
| 17 | Safe positioning |
| 18 | Forstå hvornår man skal intervenere (kende forskel på hosteinsufficiens eller ej) |
| 19 | Heimlich/thorax-kompressioner |
| 20 | Slag i ryggen |
| 21 | Kalde hjælp ved behov |
| 22 | starte genoplivning ved bevidstløshed |
| 23 | Overall adherence to algorithm |
| 24 | Correct evaluation of consciousness |
| 25 | Able to distinguish between effective/ineffective cough |
| 26 | calls for help |
| 27 | stays with conscious child |
| 28 | number of backblows/adominal/chest thrusts w sufficient force per cycle |
| 29 | positions child to utilize gravity |
| 30 | correct change in algorithm with loss of consiousness |
| 31 | learn the signs: cough followed by |
| 32 | inspiratory stridor |
| 33 | retractions |
| 34 | aphonia |
| 35 | hoarse voice |
| 36 | skin colour (blue, grey, pale) |
| 37 | salivation |
| 38 | inspection of mouth |
| 39 | 5 slaps between shoulder blades |
| 40 | compressions over sternum |
| 41 | Heimlich |
| 42 | Awareness of situation |
| 43 | Identification of ineffective cough |
| 44 | Quality of desobstruction manoeuvres a) infant |
| 45 | idem. b) children / adolescents |
| 46 | Composite score of BLS |
| 47 | Individual items similar to BLS |
| 48 | Recognition of incomplete vs. complete airway obstruction |
| 49 | Recognition of conscious vs. unconscious victim |
| 50 | Abdominal thrust (conscious patient) of greater then 30 kg force in the correct location (avoiding ribs, liver, spleen) |
| 51 | Chest compressions of at least 30 kg force (unconscious patient) |
| 52 | Recovery position for the spontaneously breathing patient |
| 53 | Activation of Emergency Medical System (Call 911?) |
| 54 | opening the airway and observing the oropharynx |
| 55 | Assessment of breathing |
| 56 | Heimlich maneuver |
| 57 | Sufficient force of back blows |
| 58 | NOT doing blind finger sweep of mouth |
| 59 | Determining need for CPR if airway obstruction results in cardiopulmonary arrest |
| 60 | Determining need for intervention |
| 61 | Should the lay person be tested on an actor or on a manikin? |
| 62 | What techniques should be tested? |
| 63 | Should there be three conditions tested: able to breathe (encourage to cough), difficulty breathing (remove object with combination of techniques), unconscious (start CPR) |
| 64 | Should the lay person be tested about what to do AFTER removal of the foreign object? |
| 65 | Should making a call to EMS be part of the test? |
| 66 | Should finger sweep be tested? How? |
| 67 | Responsivnes |
| 68 | Cough |
| 69 | Breathing |
| 70 | signs of life |
| 71 | back blows |
| 72 | abdominal thrusts |
| 73 | chest compressions |
| 74 | call for help |
| 75 | Check of responsiveness (e.g. AVPU) |
| 76 | Check of Coughing (effective/ineffective) |
| 77 | Back blows |
| 78 | Chest compressions |
| 79 | Abdominal trusts |
| 80 | BLS skills (see above) |
| 81 | Situation awareness (i.e. foreign body exposure/history) |
| 82 | Re-Evaluation |
| 83 | DETERMINING EFFECTIVENESS OF AIRWAY |
| 84 | FOLLOWING THE RIGHT SEQUENCE |
| 85 | BLS SEQUENCE PRIORITIES AS ABOVE |
| 86 | Encourage the child to cough and clear their airway themselves |
| 87 | Place the child into an appropriate position and apply 5 back slaps |
| 88 | Check to see if the object has cleared from the airway |
| 89 | In children - if the object has not cleared position appropriately and apply 5 abdominal thrusts |
| 90 | Check to see if the object has cleared from the airway |
| 91 | In infants (<1) - if the object has not cleared position appropriately and apply 5 chest thrusts |
| 92 | Check to see if the object has cleared from the airway |
| 93 | Call for help |
| 94 | Continue back slaps and abdominal thrusts/chest thrusts until the object clears or the patient becomes unconscious |
| 95 | If the patient becomes unconscious lie them on the floor and continue chest thrusts to clear the airway. |
| 96 | If unconscious it may be appropriate to try the full resuscitation sequence including ventilation |
| 97 | If the rescuer does not want to perform mouth-to-mouth resuscitation then they should continue with chest thrusts/compressions alone until help arrives |
| 98 | Ability to recognize a child with potential foreign body airway obstruction |
| 99 | Ability to recognize mild versus severe obstruction. |
| 100 | Ability to recognize unresponsiveness. |
| 101 | Ability to shout for help. |
| 102 | Ability to deliver repeated cycles of 5 back blows followed by 5 chest compressions in infants, if not unresponsive. |
| 103 | Ability to deliver repeated cycles of 5 subdiaphragmatic abdominal thrusts (Heimlich maneuver) followed by 5 chest compressions in children, if not unresponsive. |
| 104 | Ability to deliver chest compressions and ventilations if unresponsive, even with presence of pulse. |
| 105 | call for help |
| 106 | FBAO recognition |
| 107 | recognition cough effectiveness |
| 108 | deliver back blows |
| 109 | deliver abdominal thrusts in children |
| 110 | deliver chest thrusts in infants |
| 111 | recognition of loss of consciousness |
| 112 | start CPR when loss of consciousness |
| 113 | call 112 |
| 114 | single finger sweep |
| 115 | Recognition of obstruction needing intervention (child cyanotic/ not making sounds,and obstruction by solid foreign body) - often I see the use of these maneuvers for aspiration of liquids |
| 116 | Infant maneuver |
| 117 | Heimlich |
| 118 | Recognition of child needing CPR |
| 119 | Assessment of choking signs with effective cough |
| 120 | Assessment of choking signs without effective cough |
| 121 | Giving back blows in infants |
| 122 | Giving chest thrust in infants |
| 123 | Giving chest thrust in children |
| 124 | Giving abdominal thrust in children |
| 125 | Assessment of absence of |
| 126 | choking signs |
| 127 | Skills of cardiac arrest |
| 128 | Recognition of shocking |
| 129 | Recognition of effective and ineffective coughing |
| 130 | When to ask for help |
| 131 | How to ask for help |
| 132 | Basic knowledge of the algorithm of foreign body airway obstruction |
| 133 | Back blows in infants |
| 134 | Chest thrusts in infants |
| 135 | Back blows in children |
| 136 | Abdominal thrusts in children |
| 137 | Breath support : Mouth to mouth and nose technique / mouth to mouth technique |
| 138 | How to perform chest compressions in infants |
| 139 | How to perform chest compressions in children |
| 140 | How to reassess the infant/child |
| 141 | How to place the child in the recovery position |
| 142 | Ability to identify risk situations for FBAO and act on prevention​ |
| 143 | ​ Ability to diferentiate between effective and non-effective cough and identify correct what to do for each situation |
| 144 | ​ ​Ability to correctly perform back blows and adbominal / chest thrusts in different ages |
| 145 | ​ Ability to identify absence of response and switch to BLS algorithm accordingly​ |
| 146 | Ability to adequately transmit information to dispatch /EMS |
| 147 | ​ Ability to suspect FBAO during CPR if no ventilation is possible despite good airway positioning​ |
| 148 | Diagnosis of complete obstruction |
| 149 | Effective Abdominal thrusts |
| 150 | effective Back blows in a baby |
| 151 | Calling for help |
| 152 | Recognition ineffective coughing |
| 153 | Call EMS (if not done already) |
| 154 | Back Blows |
| 155 | Chest- or abdominal thrust |
| 156 | Recognition unconsious |
| 157 | Start BLS if unconscious |
| 158 | recognition (signs, etc.) |
| 159 | open airway |
| 160 | assessment (consciousness) |
| 161 | assessment (breathing) |
| 162 | open airway |
| 163 | simple measures (back slaps) |
| 164 | simple measures (chest/abdominal thrusts) |
| 165 | simple measures body position of the victim for simple measures (head down, fix/hold the body) |
| 166 | safety (own and the victim) |
| 167 | recognition of need of CPR |
| 168 | Establisch effective/ ineffective cough |
| 169 | Back blow technique |
| 170 | Thorac compressions |
| 171 | Heimlich manoeuvre |
| 172 | use of gravity in the flow of the technique( correct positioning) in infant and child |
| 173 | establish loss of consciousness as (imminent)loss of signs of life |
| 174 | CPR continuation without breathing check​ |
| 175 | Recognition of FBAO |
| 176 | Context dependent call for help |
| 177 | No delay in start-up of FBAO management​ |
| 178 | Evaluating FBAO management effectiveness |
| 179 | Non-technical skills (team work, task management, situation awareness, decision making)​ |
| 180 | Assess wheather cough is effective |
| 181 | Do not try to get the foreing body blindly |
| 182 | Difference between infant and children |
| 183 | Back blows and chest thrust/abdominal |
| 184 | Know when to start CPR |
| 185 | Recognize this situation |
| 186 | Use technique adequate to age of child |
| 187 | Corectitude of desobstructions maneuver |
| 188 | Recognize, when desobstruction maneuvers failed, the moment when child became unconscious and need CPR maneuver |
| 189 | Calling for help, knowing of emergency number |
